# Bis[μ-2-(2-carboxyl­atophen­yl)acetato]-κ^3^ *O* ^1^,*O* ^1′^:*O* ^2^;κ^3^ *O* ^2^:*O* ^1^,*O* ^1′^-bis­[aqua­(1,10-phenanthroline-κ^2^ *N*,*N*′)nickel(II)]

**DOI:** 10.1107/S1600536809018339

**Published:** 2009-05-23

**Authors:** Feng Li, Huifang Zeng, Zhaowei Yan, Taohai Li

**Affiliations:** a College of Chemistry, Key Laboratory of Environmentally Friendly Chemistry and Applications of the Ministry of Education, Xiangtan University, Hunan 411105, People’s Republic of China

## Abstract

The title compound, [Ni_2_(C_9_H_6_O_4_)_2_(C_12_H_8_N_2_)_2_(H_2_O)_2_], is isostructural with the Zn^II^ analogue. Each Ni^II^ atom is coordinated in a distorted octa­hedral geometry by three O atoms from two homophthalate anions, one aqua O atom and two 1,10-phenanthroline N atoms. The two Ni^II^ atoms are linked by two bridging homophthalate dianions into a centrosymmetric dinuclear unit. The dinuclear units are linked into one-dimensional ladder-like chains along [100] by O—H⋯O hydrogen bonds between the coordinated water mol­ecules and one of the O atoms of the carboxyl­atomethyl group.

## Related literature

For the Zn^II^ analogue, see: He *et al.* (2006 ▶); Sun (2006 ▶).
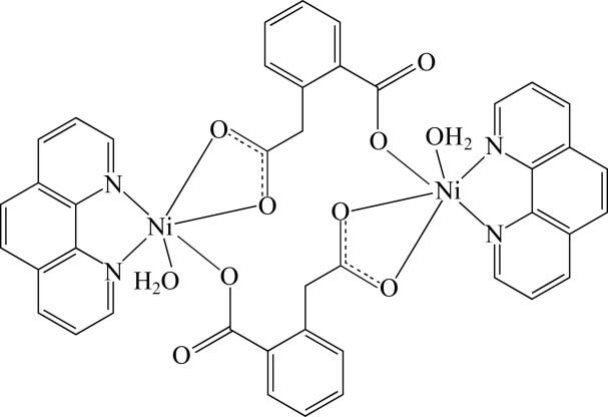


## Experimental

### 

#### Crystal data

[Ni_2_(C_9_H_6_O_4_)_2_(C_12_H_8_N_2_)_2_(H_2_O)_2_]
*M* *_r_* = 870.14
Monoclinic, 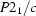

*a* = 8.819 (3) Å
*b* = 19.432 (6) Å
*c* = 12.898 (3) Å
β = 122.900 (17)°
*V* = 1855.8 (10) Å^3^
*Z* = 2
Mo *K*α radiation
μ = 1.08 mm^−1^
*T* = 173 K
0.45 × 0.40 × 0.20 mm

#### Data collection

Rigaku Mercury70 CCD diffractometer
Absorption correction: multi-scan (*CrystalClear*; Molecular Structure Corporation & Rigaku, 2001 ▶) *T*_min_ = 0.618, *T*_max_ = 0.806
14201 measured reflections
4232 independent reflections
3900 reflections with *I* > 2σ(*I*)
*R*_int_ = 0.021

#### Refinement

*R*[*F* ^2^ > 2σ(*F* ^2^)] = 0.032
*wR*(*F* ^2^) = 0.083
*S* = 1.05
4232 reflections
262 parameters
H-atom parameters constrained
Δρ_max_ = 0.43 e Å^−3^
Δρ_min_ = −0.35 e Å^−3^

### 

Data collection: *CrystalClear* (Molecular Structure Corporation & Rigaku, 2001 ▶); cell refinement: *CrystalClear*; data reduction: *CrystalClear*; program(s) used to solve structure: *SHELXS97* (Sheldrick, 2008 ▶); program(s) used to refine structure: *SHELXL97* (Sheldrick, 2008 ▶); molecular graphics: *SHELXTL* (Sheldrick, 2008 ▶); software used to prepare material for publication: *SHELXL97*.

## Supplementary Material

Crystal structure: contains datablocks I, global. DOI: 10.1107/S1600536809018339/bi2368sup1.cif

Structure factors: contains datablocks I. DOI: 10.1107/S1600536809018339/bi2368Isup2.hkl

Additional supplementary materials:  crystallographic information; 3D view; checkCIF report

## Figures and Tables

**Table 1:** Hydrogen-bond geometry (Å, °)

*D*—H⋯*A*	*D*—H	H⋯*A*	*D*⋯*A*	*D*—H⋯*A*
O1*W*—H1*WA*⋯O1^i^	0.84	1.87	2.653 (2)	155
O1*W*—H1*WB*⋯O4^ii^	0.83	1.88	2.7108 (17)	175

Symmetry codes: (i) 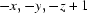; (ii) 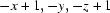.

**Fig. 1.**
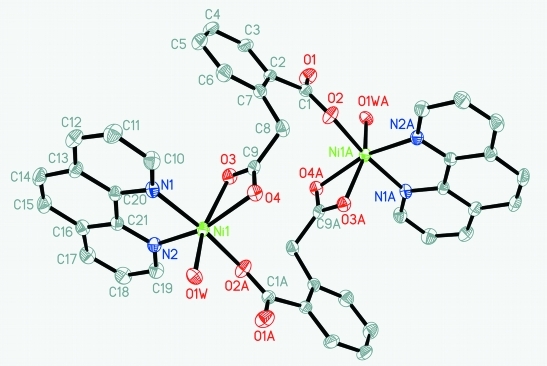
Molecular unit showing displacement ellipsoids drawn at the 30% probability level. H atoms are ommited. Symetry code: (A) -x, -y, 1 -z.

**Fig. 2.**
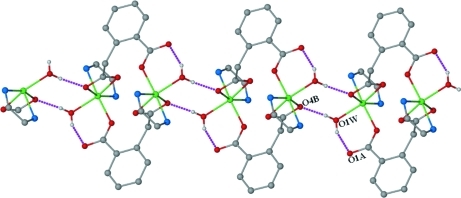
One-dimensional ladder-like chain formed along [100] by O—H···O hydrogen bonding (dashed lines). Part of the phen ligand and all H atoms that bonded to C atoms are omitted for clarity.

## supplementary crystallographic information

### Comment

The title compound is isostructural with its Zn^II^ analogue (He *et al.*, 2006; Sun, 2006). The asymmetric unit consitsts of one Ni^II^ atom, one 1,10-phenanthroline (phen) ligan, one homophthalate dianion (hpht^2-^) and a coordinated water molecule. The Ni^II^ atom is six-coordinated by two N atoms from phen and four O atoms, three from two hpht^2-^ anions and one from coordinated H_2_O, in a distorted octahedron coordination geometry (Table 1). In the hpht^2-^ ligand, the carboxylate group coordinates in a monodentate manner to Ni^II^ while the ethylcarboxylate group coordinates in a bidentate manner to another Ni^II^ atom. Two hpht^2-^ ions link two Ni^II^ atoms to form a dinuclear complex across a centre of inversion. Such units are linked to form one-dimensional ladder-like chains along [100] by O—H···O hydrogen bonds from the coordinated water molecule to one of ethylcarboxyl O atoms. π–π interactions are formed between phen units in adjacent chains.

### Experimental

Homophthalic acid (H_2_hpht; 0.0275 g, 0.15 mmol), 1,10-phenanthroline (phen; 0.030 g, 0.15 mmol) and Ni(NO_3_)_2_.6H_2_O (0.044 g, 0.15 mmol) were put in 10 ml distilled H_2_O and the pH was adjusted to about 4.2 by addind dilute NaOH aqueous solution. The mixture was sealed in a 25 ml Teflon-lined autoclave and heated to 433 K for 3 days, then slowly cooled to room temperature. Red prism crystals were collected and washed with distilled water (yield: 51%)

### Refinement

H atoms bound to C atoms were placed in calculated positions (C—H = 0.95–0.99 Å) and allowed to ride during refinement with *U*_iso_(H) = 1.2*U*_eq_(C). The H atoms on the water molecule were located from difference Fourier maps and allowed to ride in their as-found positions with constrained *U*_iso_ values.

### Crystal data

**Table:**

[Ni_2_(C_9_H_6_O_4_)_2_(C_12_H_8_N_2_)_2_(H_2_O)_2_]	*F*(000) = 896
*M**_r_* = 870.14	*D*_x_ = 1.557 Mg m^−^^3^
Monoclinic, *P*2_1_/*c*	Mo *K*α radiation, λ = 0.71073 Å
Hall symbol: -P 2ybc	Cell parameters from 5071 reflections
*a* = 8.819 (3) Å	θ = 3.1–27.5°
*b* = 19.432 (6) Å	µ = 1.08 mm^−^^1^
*c* = 12.898 (3) Å	*T* = 173 K
β = 122.900 (17)°	Prism, green
*V* = 1855.8 (10) Å^3^	0.45 × 0.40 × 0.20 mm
*Z* = 2	

### Data collection

**Table:**

Rigaku Mercury70 CCD diffractometer	4232 independent reflections
Radiation source: fine-focus sealed tube	3900 reflections with *I* > 2σ(*I*)
graphite	*R*_int_ = 0.021
φ scans	θ_max_ = 27.5°, θ_min_ = 3.1°
Absorption correction: multi-scan (*CrystalClear*; Molecular Structure Corporation & Rigaku, 2001)	*h* = −11→10
*T*_min_ = 0.618, *T*_max_ = 0.806	*k* = −25→25
14201 measured reflections	*l* = −8→16

### Refinement

**Table:**

Refinement on *F*^2^	Primary atom site location: structure-invariant direct methods
Least-squares matrix: full	Secondary atom site location: difference Fourier map
*R*[*F*^2^ > 2σ(*F*^2^)] = 0.032	Hydrogen site location: inferred from neighbouring sites
*wR*(*F*^2^) = 0.083	H-atom parameters constrained
*S* = 1.05	*w* = 1/[σ^2^(*F*_o_^2^) + (0.0409*P*)^2^ + 0.7079*P*] where *P* = (*F*_o_^2^ + 2*F*_c_^2^)/3
4232 reflections	(Δ/σ)_max_ = 0.001
262 parameters	Δρ_max_ = 0.43 e Å^−^^3^
0 restraints	Δρ_min_ = −0.35 e Å^−^^3^

### Special details

**Table:**

Geometry. All e.s.d.'s (except the e.s.d. in the dihedral angle between two l.s. planes) are estimated using the full covariance matrix. The cell e.s.d.'s are taken into account individually in the estimation of e.s.d.'s in distances, angles and torsion angles; correlations between e.s.d.'s in cell parameters are only used when they are defined by crystal symmetry. An approximate (isotropic) treatment of cell e.s.d.'s is used for estimating e.s.d.'s involving l.s. planes.
Refinement. Refinement of *F*^2^ against ALL reflections. The weighted *R*-factor *wR* and goodness of fit *S* are based on *F*^2^, conventional *R*-factors *R* are based on *F*, with *F* set to zero for negative *F*^2^. The threshold expression of *F*^2^ > σ(*F*^2^) is used only for calculating *R*-factors(gt) *etc*. and is not relevant to the choice of reflections for refinement. *R*-factors based on *F*^2^ are statistically about twice as large as those based on *F*, and *R*- factors based on ALL data will be even larger.

### Fractional atomic coordinates and isotropic or equivalent isotropic displacement parameters (Å^2^)

**Table:**

	*x*	*y*	*z*	*U*_iso_*/*U*_eq_	
Ni1	0.44081 (3)	−0.039291 (11)	0.673702 (19)	0.03024 (8)	
O1	−0.33605 (19)	0.17448 (7)	0.51356 (14)	0.0502 (4)	
N1	0.6408 (2)	0.03318 (7)	0.77780 (14)	0.0345 (3)	
C1	−0.2231 (2)	0.15639 (8)	0.48860 (15)	0.0314 (3)	
O2	−0.25662 (17)	0.11526 (7)	0.40350 (12)	0.0435 (3)	
N2	0.52689 (19)	−0.07298 (7)	0.84877 (13)	0.0340 (3)	
C2	−0.0352 (2)	0.18683 (8)	0.56453 (15)	0.0304 (3)	
O3	0.23654 (17)	0.03212 (6)	0.64245 (12)	0.0364 (3)	
C3	−0.0064 (3)	0.23714 (9)	0.65075 (17)	0.0400 (4)	
H3	−0.1037	0.2502	0.6584	0.048*	
O4	0.31651 (16)	0.02133 (6)	0.50972 (11)	0.0348 (3)	
C4	0.1591 (3)	0.26839 (10)	0.72498 (19)	0.0497 (5)	
H4	0.1761	0.3015	0.7847	0.060*	
C5	0.2996 (3)	0.25140 (11)	0.7122 (2)	0.0534 (5)	
H5	0.4133	0.2736	0.7613	0.064*	
C6	0.2739 (3)	0.20201 (11)	0.6275 (2)	0.0485 (5)	
H6	0.3715	0.1908	0.6190	0.058*	
C7	0.1087 (2)	0.16760 (9)	0.55342 (16)	0.0340 (4)	
C8	0.1050 (2)	0.11111 (10)	0.47180 (16)	0.0389 (4)	
H8A	−0.0204	0.0944	0.4169	0.047*	
H8B	0.1451	0.1299	0.4192	0.047*	
C9	0.2252 (2)	0.05161 (9)	0.54613 (16)	0.0306 (3)	
C10	0.6981 (3)	0.08452 (10)	0.74036 (19)	0.0448 (4)	
H10	0.6441	0.0910	0.6542	0.054*	
C11	0.8343 (3)	0.12948 (11)	0.8218 (2)	0.0521 (5)	
H11	0.8714	0.1659	0.7913	0.062*	
C12	0.9141 (3)	0.12072 (11)	0.9459 (2)	0.0483 (5)	
H12	1.0071	0.1511	1.0024	0.058*	
C13	0.8583 (2)	0.06661 (10)	0.99009 (17)	0.0390 (4)	
C14	0.9333 (3)	0.05242 (11)	1.11804 (19)	0.0475 (5)	
H14	1.0284	0.0806	1.1788	0.057*	
C15	0.8722 (3)	0.00016 (11)	1.15414 (17)	0.0472 (5)	
H15	0.9228	−0.0073	1.2397	0.057*	
C16	0.7318 (3)	−0.04433 (9)	1.06539 (17)	0.0386 (4)	
C17	0.6650 (3)	−0.10109 (11)	1.09668 (18)	0.0473 (5)	
H17	0.7111	−0.1112	1.1808	0.057*	
C18	0.5337 (3)	−0.14141 (11)	1.00544 (19)	0.0496 (5)	
H18	0.4877	−0.1798	1.0256	0.060*	
C19	0.4669 (3)	−0.12587 (10)	0.88166 (18)	0.0428 (4)	
H19	0.3752	−0.1543	0.8190	0.051*	
C20	0.7199 (2)	0.02413 (9)	0.90086 (16)	0.0323 (3)	
C21	0.6584 (2)	−0.03289 (8)	0.94044 (16)	0.0320 (3)	
O1W	0.60875 (16)	−0.09246 (6)	0.63816 (12)	0.0362 (3)	
H1WA	0.5459	−0.1231	0.5868	0.060*	
H1WB	0.6287	−0.0686	0.5935	0.038*	

### Atomic displacement parameters (Å^2^)

**Table:**

	*U*^11^	*U*^22^	*U*^33^	*U*^12^	*U*^13^	*U*^23^
Ni1	0.02846 (13)	0.03614 (13)	0.02636 (13)	−0.00226 (8)	0.01504 (10)	−0.00119 (8)
O1	0.0441 (8)	0.0540 (8)	0.0621 (9)	−0.0049 (6)	0.0350 (7)	−0.0166 (7)
N1	0.0306 (7)	0.0422 (8)	0.0293 (7)	−0.0014 (6)	0.0153 (6)	0.0026 (6)
C1	0.0342 (8)	0.0300 (7)	0.0306 (8)	−0.0002 (6)	0.0181 (7)	0.0024 (6)
O2	0.0400 (7)	0.0534 (8)	0.0438 (8)	−0.0164 (6)	0.0272 (6)	−0.0193 (6)
N2	0.0342 (7)	0.0387 (7)	0.0302 (7)	−0.0011 (6)	0.0181 (6)	0.0004 (6)
C2	0.0357 (8)	0.0257 (7)	0.0281 (8)	−0.0009 (6)	0.0162 (7)	0.0020 (6)
O3	0.0403 (7)	0.0432 (6)	0.0314 (6)	0.0010 (5)	0.0231 (6)	−0.0005 (5)
C3	0.0523 (11)	0.0305 (8)	0.0396 (10)	−0.0026 (7)	0.0265 (9)	−0.0046 (7)
O4	0.0327 (6)	0.0443 (6)	0.0325 (6)	0.0047 (5)	0.0211 (5)	0.0013 (5)
C4	0.0623 (13)	0.0335 (9)	0.0437 (11)	−0.0103 (9)	0.0226 (10)	−0.0102 (8)
C5	0.0465 (11)	0.0453 (10)	0.0509 (12)	−0.0151 (9)	0.0151 (10)	−0.0049 (9)
C6	0.0356 (10)	0.0530 (11)	0.0516 (12)	−0.0046 (8)	0.0203 (9)	−0.0003 (10)
C7	0.0339 (8)	0.0347 (8)	0.0304 (8)	0.0009 (6)	0.0154 (7)	0.0032 (7)
C8	0.0343 (9)	0.0526 (10)	0.0292 (9)	0.0098 (8)	0.0169 (8)	0.0005 (8)
C9	0.0259 (8)	0.0381 (8)	0.0276 (8)	−0.0046 (6)	0.0145 (7)	−0.0063 (7)
C10	0.0437 (10)	0.0504 (10)	0.0389 (10)	−0.0055 (8)	0.0214 (9)	0.0057 (8)
C11	0.0544 (12)	0.0495 (11)	0.0546 (13)	−0.0147 (9)	0.0311 (11)	−0.0018 (10)
C12	0.0457 (11)	0.0481 (11)	0.0502 (12)	−0.0130 (9)	0.0254 (10)	−0.0132 (9)
C13	0.0345 (9)	0.0448 (9)	0.0363 (10)	−0.0027 (7)	0.0184 (8)	−0.0095 (8)
C14	0.0440 (11)	0.0577 (12)	0.0330 (10)	−0.0044 (9)	0.0158 (9)	−0.0140 (9)
C15	0.0497 (11)	0.0617 (12)	0.0252 (9)	0.0009 (9)	0.0170 (8)	−0.0058 (8)
C16	0.0401 (10)	0.0478 (10)	0.0302 (9)	0.0060 (8)	0.0206 (8)	0.0002 (7)
C17	0.0564 (12)	0.0577 (12)	0.0330 (10)	0.0054 (10)	0.0276 (10)	0.0077 (9)
C18	0.0598 (13)	0.0514 (11)	0.0442 (11)	−0.0019 (9)	0.0325 (10)	0.0096 (9)
C19	0.0454 (10)	0.0456 (10)	0.0392 (10)	−0.0060 (8)	0.0242 (9)	0.0023 (8)
C20	0.0274 (8)	0.0397 (8)	0.0289 (8)	0.0011 (6)	0.0148 (7)	−0.0018 (7)
C21	0.0284 (8)	0.0374 (8)	0.0308 (9)	0.0036 (6)	0.0165 (7)	−0.0021 (7)
O1W	0.0328 (6)	0.0400 (6)	0.0381 (7)	0.0055 (5)	0.0207 (6)	0.0077 (5)

### Geometric parameters (Å, °)

**Table:**

Ni1—O2^i^	2.0130 (13)	C7—C8	1.509 (2)
Ni1—O1W	2.0515 (13)	C8—C9	1.508 (2)
Ni1—N2	2.0595 (15)	C8—H8A	0.990
Ni1—N1	2.0794 (16)	C8—H8B	0.990
Ni1—O4	2.1316 (13)	C10—C11	1.393 (3)
Ni1—O3	2.1319 (14)	C10—H10	0.950
O1—C1	1.252 (2)	C11—C12	1.365 (3)
N1—C10	1.323 (2)	C11—H11	0.950
N1—C20	1.354 (2)	C12—C13	1.406 (3)
C1—O2	1.256 (2)	C12—H12	0.950
C1—C2	1.514 (2)	C13—C20	1.403 (2)
O2—Ni1^i^	2.0130 (13)	C13—C14	1.432 (3)
N2—C19	1.327 (2)	C14—C15	1.346 (3)
N2—C21	1.364 (2)	C14—H14	0.950
C2—C3	1.397 (2)	C15—C16	1.432 (3)
C2—C7	1.403 (2)	C15—H15	0.950
O3—C9	1.250 (2)	C16—C21	1.390 (3)
C3—C4	1.377 (3)	C16—C17	1.409 (3)
C3—H3	0.950	C17—C18	1.363 (3)
O4—C9	1.275 (2)	C17—H17	0.950
C4—C5	1.376 (3)	C18—C19	1.402 (3)
C4—H4	0.950	C18—H18	0.950
C5—C6	1.377 (3)	C19—H19	0.950
C5—H5	0.950	C20—C21	1.443 (2)
C6—C7	1.404 (3)	O1W—H1WA	0.837
C6—H6	0.950	O1W—H1WB	0.828
			
O2^i^—Ni1—O1W	90.38 (6)	C7—C8—H8A	109.2
O2^i^—Ni1—N2	91.55 (6)	C9—C8—H8B	109.2
O1W—Ni1—N2	101.77 (6)	C7—C8—H8B	109.2
O2^i^—Ni1—N1	171.71 (6)	H8A—C8—H8B	107.9
O1W—Ni1—N1	91.33 (6)	O3—C9—O4	120.04 (16)
N2—Ni1—N1	80.16 (6)	O3—C9—C8	120.90 (15)
O2^i^—Ni1—O4	94.16 (6)	O4—C9—C8	119.06 (15)
O1W—Ni1—O4	95.81 (5)	N1—C10—C11	122.82 (19)
N2—Ni1—O4	161.47 (5)	N1—C10—H10	118.6
N1—Ni1—O4	93.73 (6)	C11—C10—H10	118.6
O2^i^—Ni1—O3	90.68 (6)	C12—C11—C10	119.36 (19)
O1W—Ni1—O3	157.53 (5)	C12—C11—H11	120.3
N2—Ni1—O3	100.64 (5)	C10—C11—H11	120.3
N1—Ni1—O3	90.83 (6)	C11—C12—C13	119.84 (18)
O4—Ni1—O3	61.73 (5)	C11—C12—H12	120.1
C10—N1—C20	118.06 (16)	C13—C12—H12	120.1
C10—N1—Ni1	129.31 (13)	C20—C13—C12	116.59 (17)
C20—N1—Ni1	112.64 (11)	C20—C13—C14	118.91 (18)
O1—C1—O2	124.09 (16)	C12—C13—C14	124.50 (18)
O1—C1—C2	117.91 (15)	C15—C14—C13	121.51 (18)
O2—C1—C2	118.00 (15)	C15—C14—H14	119.2
C1—O2—Ni1^i^	129.98 (11)	C13—C14—H14	119.2
C19—N2—C21	117.66 (16)	C14—C15—C16	120.91 (18)
C19—N2—Ni1	128.56 (13)	C14—C15—H15	119.5
C21—N2—Ni1	113.73 (11)	C16—C15—H15	119.5
C3—C2—C7	118.98 (16)	C21—C16—C17	116.94 (18)
C3—C2—C1	116.77 (15)	C21—C16—C15	119.12 (17)
C7—C2—C1	124.25 (15)	C17—C16—C15	123.92 (18)
C9—O3—Ni1	89.34 (10)	C18—C17—C16	119.59 (18)
C4—C3—C2	121.85 (18)	C18—C17—H17	120.2
C4—C3—H3	119.1	C16—C17—H17	120.2
C2—C3—H3	119.1	C17—C18—C19	119.58 (19)
C9—O4—Ni1	88.68 (10)	C17—C18—H18	120.2
C5—C4—C3	119.61 (19)	C19—C18—H18	120.2
C5—C4—H4	120.2	N2—C19—C18	122.49 (18)
C3—C4—H4	120.2	N2—C19—H19	118.8
C4—C5—C6	119.43 (19)	C18—C19—H19	118.8
C4—C5—H5	120.3	N1—C20—C13	123.33 (16)
C6—C5—H5	120.3	N1—C20—C21	117.46 (15)
C5—C6—C7	122.33 (19)	C13—C20—C21	119.21 (16)
C5—C6—H6	118.8	N2—C21—C16	123.73 (16)
C7—C6—H6	118.8	N2—C21—C20	115.96 (15)
C2—C7—C6	117.74 (17)	C16—C21—C20	120.31 (16)
C2—C7—C8	125.94 (16)	Ni1—O1W—H1WA	106.6
C6—C7—C8	116.28 (16)	Ni1—O1W—H1WB	109.0
C9—C8—C7	111.88 (14)	H1WA—O1W—H1WB	98.5
C9—C8—H8A	109.2		
			
O1W—Ni1—N1—C10	76.39 (17)	C2—C7—C8—C9	−112.08 (19)
N2—Ni1—N1—C10	178.11 (18)	C6—C7—C8—C9	65.6 (2)
O4—Ni1—N1—C10	−19.52 (17)	Ni1—O3—C9—O4	4.62 (16)
O3—Ni1—N1—C10	−81.24 (17)	Ni1—O3—C9—C8	−175.03 (14)
O1W—Ni1—N1—C20	−103.35 (12)	Ni1—O4—C9—O3	−4.62 (16)
N2—Ni1—N1—C20	−1.63 (12)	Ni1—O4—C9—C8	175.04 (14)
O4—Ni1—N1—C20	160.74 (12)	C7—C8—C9—O3	38.4 (2)
O3—Ni1—N1—C20	99.02 (12)	C7—C8—C9—O4	−141.28 (16)
O1—C1—O2—Ni1^i^	−26.5 (3)	C20—N1—C10—C11	−0.5 (3)
C2—C1—O2—Ni1^i^	154.47 (12)	Ni1—N1—C10—C11	179.76 (15)
O2^i^—Ni1—N2—C19	−0.48 (16)	N1—C10—C11—C12	0.3 (3)
O1W—Ni1—N2—C19	−91.19 (16)	C10—C11—C12—C13	0.0 (3)
N1—Ni1—N2—C19	179.50 (17)	C11—C12—C13—C20	−0.2 (3)
O4—Ni1—N2—C19	107.5 (2)	C11—C12—C13—C14	179.5 (2)
O3—Ni1—N2—C19	90.51 (16)	C20—C13—C14—C15	−1.0 (3)
O2^i^—Ni1—N2—C21	−177.86 (12)	C12—C13—C14—C15	179.2 (2)
O1W—Ni1—N2—C21	91.43 (12)	C13—C14—C15—C16	1.4 (3)
N1—Ni1—N2—C21	2.12 (11)	C14—C15—C16—C21	−0.1 (3)
O4—Ni1—N2—C21	−69.8 (2)	C14—C15—C16—C17	178.2 (2)
O3—Ni1—N2—C21	−86.87 (12)	C21—C16—C17—C18	−0.2 (3)
O1—C1—C2—C3	−4.9 (2)	C15—C16—C17—C18	−178.54 (19)
O2—C1—C2—C3	174.24 (16)	C16—C17—C18—C19	0.0 (3)
O1—C1—C2—C7	175.03 (16)	C21—N2—C19—C18	−0.1 (3)
O2—C1—C2—C7	−5.9 (2)	Ni1—N2—C19—C18	−177.39 (15)
O2^i^—Ni1—O3—C9	−97.07 (10)	C17—C18—C19—N2	0.2 (3)
O1W—Ni1—O3—C9	−4.43 (19)	C10—N1—C20—C13	0.4 (3)
N2—Ni1—O3—C9	171.22 (10)	Ni1—N1—C20—C13	−179.87 (13)
N1—Ni1—O3—C9	91.08 (11)	C10—N1—C20—C21	−178.83 (16)
O4—Ni1—O3—C9	−2.71 (9)	Ni1—N1—C20—C21	0.94 (19)
C7—C2—C3—C4	0.0 (3)	C12—C13—C20—N1	0.0 (3)
C1—C2—C3—C4	179.96 (16)	C14—C13—C20—N1	−179.76 (17)
O2^i^—Ni1—O4—C9	91.19 (10)	C12—C13—C20—C21	179.15 (16)
O1W—Ni1—O4—C9	−178.00 (10)	C14—C13—C20—C21	−0.6 (3)
N2—Ni1—O4—C9	−16.4 (2)	C19—N2—C21—C16	−0.2 (3)
N1—Ni1—O4—C9	−86.27 (10)	Ni1—N2—C21—C16	177.54 (13)
O3—Ni1—O4—C9	2.66 (9)	C19—N2—C21—C20	−179.95 (15)
C2—C3—C4—C5	1.9 (3)	Ni1—N2—C21—C20	−2.26 (18)
C3—C4—C5—C6	−1.7 (3)	C17—C16—C21—N2	0.3 (3)
C4—C5—C6—C7	−0.4 (3)	C15—C16—C21—N2	178.70 (17)
C3—C2—C7—C6	−2.1 (2)	C17—C16—C21—C20	−179.90 (16)
C1—C2—C7—C6	178.01 (16)	C15—C16—C21—C20	−1.5 (3)
C3—C2—C7—C8	175.58 (16)	N1—C20—C21—N2	0.9 (2)
C1—C2—C7—C8	−4.3 (3)	C13—C20—C21—N2	−178.35 (15)
C5—C6—C7—C2	2.3 (3)	N1—C20—C21—C16	−178.93 (16)
C5—C6—C7—C8	−175.61 (19)	C13—C20—C21—C16	1.8 (2)

Symmetry codes: (i) −*x*, −*y*, −*z*+1.

### Hydrogen-bond geometry (Å, °)

**Table:**

*D*—H···*A*	*D*—H	H···*A*	*D*···*A*	*D*—H···*A*
O1W—H1WA···O1^i^	0.84	1.87	2.653 (2)	155
O1W—H1WB···O4^ii^	0.83	1.88	2.7108 (17)	175

Symmetry codes: (i) −*x*, −*y*, −*z*+1; (ii) −*x*+1, −*y*, −*z*+1.
